# Digital Game-Based Phonics Instruction Promotes Print Knowledge in Pre-Readers at Cognitive Risk for Dyslexia

**DOI:** 10.3389/fpsyg.2021.720548

**Published:** 2021-09-08

**Authors:** Femke Vanden Bempt, Maria Economou, Shauni Van Herck, Jolijn Vanderauwera, Toivo Glatz, Maaike Vandermosten, Jan Wouters, Pol Ghesquière

**Affiliations:** ^1^Parenting and Special Education Research Unit, Faculty of Psychology and Educational Sciences, KU Leuven, Leuven, Belgium; ^2^Research Group ExpORL, Department of Neurosciences, KU Leuven, Leuven, Belgium; ^3^Psychological Sciences Research Institute, Université Catholique de Louvain, Ottignies-Louvain-la-Neuve, Belgium; ^4^Institute of Neuroscience, Université Catholique de Louvain, Ottignies-Louvain-la-Neuve, Belgium; ^5^Institute of Public Health, Charité – Universitätsmedizin Berlin, Freie Universität Berlin and Humboldt-Universität zu Berlin, Berlin, Germany

**Keywords:** dyslexia, game-based intervention, pre-readers, phonics instruction, preventive

## Abstract

Dyslexia is targeted most effectively when (1) interventions are provided preventively, before the onset of reading instruction, and (2) remediation programs combine letter-sound training with phoneme blending. Given the growing potential of technology in educational contexts, there has been a considerable increase of letter-sound trainings embedded in digital serious games. One such intervention is GraphoGame. Yet, current evidence on the preventive impact of GraphoGame is limited by the lack of adaptation of the original learning content to the skills of pre-readers, short training duration, and a restricted focus on explicitly trained skills. Therefore, the current study aims at investigating the impact of a preventive, and pre-reading adapted GraphoGame training (i.e., GraphoGame-Flemish, GG-FL) on explicitly trained skills and non-specifically trained phonological and language abilities. Following a large-scale screening (*N* = 1225), the current study included 88 pre-reading kindergarteners at cognitive risk for dyslexia who were assigned to three groups training either with GG-FL (*n* = 31), an active control game (*n* = 29), or no game (*n* = 28). Before and after the 12-week intervention, a variety of reading-related skills were assessed. Moreover, receptive letter knowledge and phonological awareness were measured every three weeks during the intervention period. Results revealed significantly larger improvements in the GG-FL group on explicitly trained skills, i.e., letter knowledge and word decoding, without finding transfer-effects to untrained phonological and language abilities. Our findings imply a GG-FL-driven head start on early literacy skills in at-risk children. A follow-up study should uncover the long-term impact and the ability of GG-FL to prevent actual reading failure.

## Introduction

Learning to read with understanding requires intact word decoding, language, and cognition (Storch and Whitehurst, 2002; Goff et al., 2005; Partanen and Siegel, 2014; Horowitz-Kraus et al., 2017; McArthur and Castles, 2017). Despite this multi-componential complexity, most children learn to read fluently with comprehension throughout the primary school grades. However, around 3–7% of the population suffers from developmental dyslexia (henceforth dyslexia) (Gersons-Wolfensberger and Ruijssenaars, 1997), a specific learning disability characterized by severe and persistent shortcomings in accurate and fluent word decoding and/or spelling (Lyon et al., 2003). The experienced difficulties in dyslexia are generally worse than expected based on the amount and quality of instruction and the readers' physical, neurological, and intellectual abilities (Lyon et al., 2003; American Psychiatric Association, 2014).

Given the relatively high prevalence of dyslexia and the potential detrimental consequences on educational and socio-emotional development (Valås, 1999; Undheim, 2009), much research has been conducted on the cause and the most successful treatment of this specific learning disorder. As for the cause, it has been proposed that the process of decoding is constrained by compromised phonological abilities (Vellutino et al., 2004). Generally, phonological abilities include three subcomponents: phonological awareness (PA), verbal short-term memory (VSTM), and rapid automatized naming (RAN) (Wagner and Torgesen, 1987). PA is the ability to distinguish and manipulate speech sounds in the spoken language domain. It is the phonological skill that is most closely linked to reading acquisition since it lays the foundation for grapheme-phoneme mapping (Hulme et al., 2002; Castles and Coltheart, 2004). VSTM involves storing, ordering, and recalling auditory information via a phonologically-based memory system. VSTM particularly plays a critical role in the early stages of reading (Martinez Perez et al., 2012; Cunningham et al., 2021), when readers still decode each grapheme separately, store the isolated successive phonemes temporarily in their short-term memory, and blend them together to form the whole word form. Finally, RAN involves the ability to couple symbols to their auditory representation by rapidly and efficiently evoking the phonological code from the long-term memory, a process which is also relied on when reading words. Although top-down language knowledge is also involved as a facilitator in learning to decode print (Share, 1999), many studies across different languages and orthographies particularly reported phonological shortcomings in readers with dyslexia (Nittrouer, 1999; De Jong and Van Der Leij, 2003), but also in pre-readers at risk for reading failure (Catts et al., 2001; Boets et al., 2010; Lyytinen et al., 2015). The abovementioned three-part unitary phonological construct has been heavily debated because of several reasons. First, RAN and PA appear to predict different aspects of reading ability, i.e., reading speed and accuracy respectively (Georgiou et al., 2008; Poulsen et al., 2015) and children who exhibit both RAN and PA difficulties are likely to experience more severe reading problems compared to children with difficulties in only one skill (Papadopoulos et al., 2009). These findings suggest that PA and RAN are different constructs. Secondly, as for VSTM, the specific process of ordering and maintaining phonemes has been considered as a separate reading-related precursor different from phonological processing *per se* (Martinez Perez et al., 2012). As for the treatment of dyslexia, supporting the phonological deficit theory, a phonics-based approach, including systematic and explicit instruction of letter-sound relations combined with phoneme blending exercises, appeared to be most successful in tackling the decoding deficits readers with dyslexia are faced with (National Institute of Child Health Human Development, 2000; Snowling and Hulme, 2011; Galuschka et al., 2014).

Despite the well-established efficacy of phonics-based interventions, readers with dyslexia usually do not reach the reading level of their typically developing peers (Torgesen et al., 1997; Ferrer et al., 2015) and the achievement gap even tends to widen over time (Vaughn et al., 2009). A possible explanatory factor for this achievement gap concerns the dyslexia paradox (Ozernov-Palchik and Gaab, 2016). This paradox encompasses the fact that in current clinical practice, reading interventions are usually only provided after defining a clear reading and/or spelling deficit (usually from second grade onwards), while early preventive literacy interventions that are offered before the onset of reading and spelling problems tend to be more effective than reading remediation at a later age (Wanzek and Vaughn, 2007; Lovett et al., 2017). Thus, by the time interventional trajectories usually start up, the most effective period for therapy has passed. The dyslexia paradox thus emphasizes the need for preventive phonics-based interventions in at-risk pre-reading children who already exhibit low performance on important precursors of reading ability.

Providing phonics-based preventive interventions in all at-risk children, however, is often practically and economically unfeasible. Moreover, motivating young pre-reading children for these types of interventions is challenging, especially when they already perform low on reading precursors and when they have not yet received any type of reading instruction before. Fortunately, digital gaming interventions offer a solution to these challenges. For instance, phonics-based digital games can be easily distributed at a large scale, thereby by-passing feasibility issues. In addition, digital games generally carry the possibility to (1) provide age-appropriate and attractive audiovisual stimuli, (2) give immediate feedback, and (3) adapt the difficulty-level to obtain a tailor-made trade-off between the level of challenge and the player's skills. These game features are important prerequisites to obtain a feeling of enjoyment (Sweetser and Wyeth, 2005), which enhances motivation (Hsi, 2007; van de Ven et al., 2017) and fosters learning (Shute et al., 2009).

Most kindergarten phonics-focused interventions, using a real-life (Schneider et al., 2000; Elbro and Petersen, 2004; Bowyer-Crane et al., 2008; Van Otterloo and Van Der Leij, 2009) or a digital game-based approach (Regtvoort and Van Der Leij, 2007; Macaruso and Walker, 2008; Kegel et al., 2009; Kegel and Bus, 2012; Savage et al., 2013; Piquette et al., 2014) generally yielded direct short-term effects on trained skills, e.g., word decoding and letter knowledge (LK). Findings of intervention-driven PA improvements were rather mixed. These results are somewhat surprising since all aforementioned studies contained a PA component. Moreover, a reciprocal influence of explicit reading instruction on PA has also been widely established (Ziegler and Goswami, 2005). Some researchers claim that PA is simply a skill that is difficult to improve by training (Hatcher et al., 1994). Alternatively, although contradicted (Duncan et al., 2013), another explanation for the mixed findings across studies presumably relates to the claim that the impact of reading development on PA emerges more slowly in opaque than transparent orthographies, given their irregular grapheme-phoneme mappings (Goswami et al., 2001). Evidence exists that reading development not only influences PA, but also other phonological abilities, e.g., rapid naming (RAN) (Peterson et al., 2018), verbal short-term memory (VSTM) (Nation and Hulme, 2011; Demoulin and Kolinsky, 2016), and broader language skills (Kolinsky, 2015; Hulme et al., 2019). None of the aforementioned studies which explored these relationships however, determined generalization (Schneider et al., 2000; Elbro and Petersen, 2004; Regtvoort and Van Der Leij, 2007; Bowyer-Crane et al., 2008; Van Otterloo and Van Der Leij, 2009). These findings raise the idea that phonics-based intervention effects in pre-readers are rather training specific.

An example of a widely evaluated game-based phonics-focused intervention program, is GraphoGame (Richardson and Lyytinen, 2014). This game-based intervention, which mainly focuses on explicit and systematic phonics instruction, already showed benefits on LK, PA, reading accuracy, fluency, and spelling across different language contexts and age groups of reading children (Richardson and Lyytinen, 2014; Ronimus and Richardson, 2014; McTigue et al., 2019). In their meta-analysis, McTigue et al. (2019) did not establish a significant influence of orthographic depth on the efficacy of GG on reading ability. The authors claimed that this was due to the precise orthographic-depth-based adaptations in the set of existing GG versions. To the best of our knowledge, two studies also evaluated the impact of GraphoGame (GG) in pre-readers, but solely on specifically trained decoding-related skills (Brem et al., 2010; Lovio et al., 2012). When compared to an active control group, which played a number knowledge game, Brem et al. (2010) found larger improvements on letter knowledge and reading skills after a GG training of 3.6 h in Swiss-German pre-readers, a subset of them at familial risk for dyslexia. However, only three children of the 32 participants managed to read more than 10 words directly after the GG training period, a finding that was not surprising given the main focus of GG on grapheme-phoneme relations. Despite the same training duration of ~three h, Lovio et al. (2012) did not demonstrate clear GraphoGame-driven effects on reading, writing, phonological awareness, and letter knowledge. However, unlike Brem et al. (2010), they included a sample of pre-reading children at cognitive risk for dyslexia, based on low pre-literacy skills in kindergarten. These findings raise the idea that the original GraphoGame-based approach is not suitable for pre-readers who already perform low on reading-related measures in kindergarten. Two possible shortcomings are discussed below. First, given the expected slower progress in pre-reading children with low pre-literacy skills (Catts et al., 2001; Boets et al., 2010), an exposure time of ~three h is possibly simply too short for children at cognitive risk to capture the content of the game. The second shortcoming concerns the general GG method instruction. To clarify, the classic version of GG starts with grapheme-phoneme mapping, auditory and visual synthesis exercises and gradually continues to train connections between syllables, whole words or pseudowords with their corresponding written language segments (Richardson and Lyytinen, 2014). However, Stanovich and Stanovich (1995) argue that awareness of the segmental structure of spoken and written language is a condition to correctly apply grapheme-phoneme conversions. This stage-based developmental approach was also adopted in the developmental reading model of Struiksma et al. (2004), which argues that optimized grapheme-phoneme connections partly result from so-called pre-reading literacy requirements, such as visual and auditory discrimination skills. Indeed, in order to correctly acquire grapho-phonemic conversion skills in alphabetic languages, the learner must be able to discriminate between all possible letter symbols (graphemes) (Woodrome and Johnson, 2009; Serniclaes, 2018) and between the set of speech sounds (phonemes) used in the spoken language of interest (Serniclaes, 2018). In line with this stage-based approach for learning to read, we hypothesize that offering grapheme-phoneme correspondence instruction from the start without a gradual build-up including preceding visual and auditory discrimination exercises is too abrupt and too difficult for children at cognitive risk.

Driven by the two aforementioned shortcomings, the current study aimed at evaluating the impact of a preventive intervention in pre-readers at cognitive risk for dyslexia, using an adapted version of GraphoGame, i.e., GraphoGame Flemish (GG-FL), with a longer training duration of 18 h compared to previous preventive GG studies (Brem et al., 2010; Lovio et al., 2012). As opposed to the classic GG versions, GG-FL provides a gradual build-up, starting with auditory and visual discrimination exercises before offering grapheme-phoneme coupling training. Although not directly tested in the current study, we believe that this GG-FL version is a more suitable tool for a pre-reading population than former GG versions. The first goal was to shed light on specifically trained decoding-related skills (LK, PA, word decoding), on which we hypothesized to find GG-FL driven influences. To extend existing literature, the second goal was to explore possible transfer effects on non-specifically trained phonological abilities (RAN and VSTM), and broader language skills (morphological awareness and receptive vocabulary) known to be influenced by literacy development. Based on the idea of specificity in kindergarten reading interventions, supported by the null-findings in previous studies, we did not expect to find any generalization in the current study. A traditional pre-post intervention study design was employed to evaluate the influence on word decoding, productive LK, RAN, VSTM, and the broader language skills. Although this pre-post intervention design type has been widely used to determine success of an intervention program (Bellotti et al., 2013), multiple intermediate assessments allow for a more precise estimation of growth parameters. Therefore, the core skills trained by GG-FL, e.g., receptive LK and PA, were assessed five times during the intervention period by means of a digital game-based tool at home.

## Materials and Methods

### Participants

**Figure 1:**
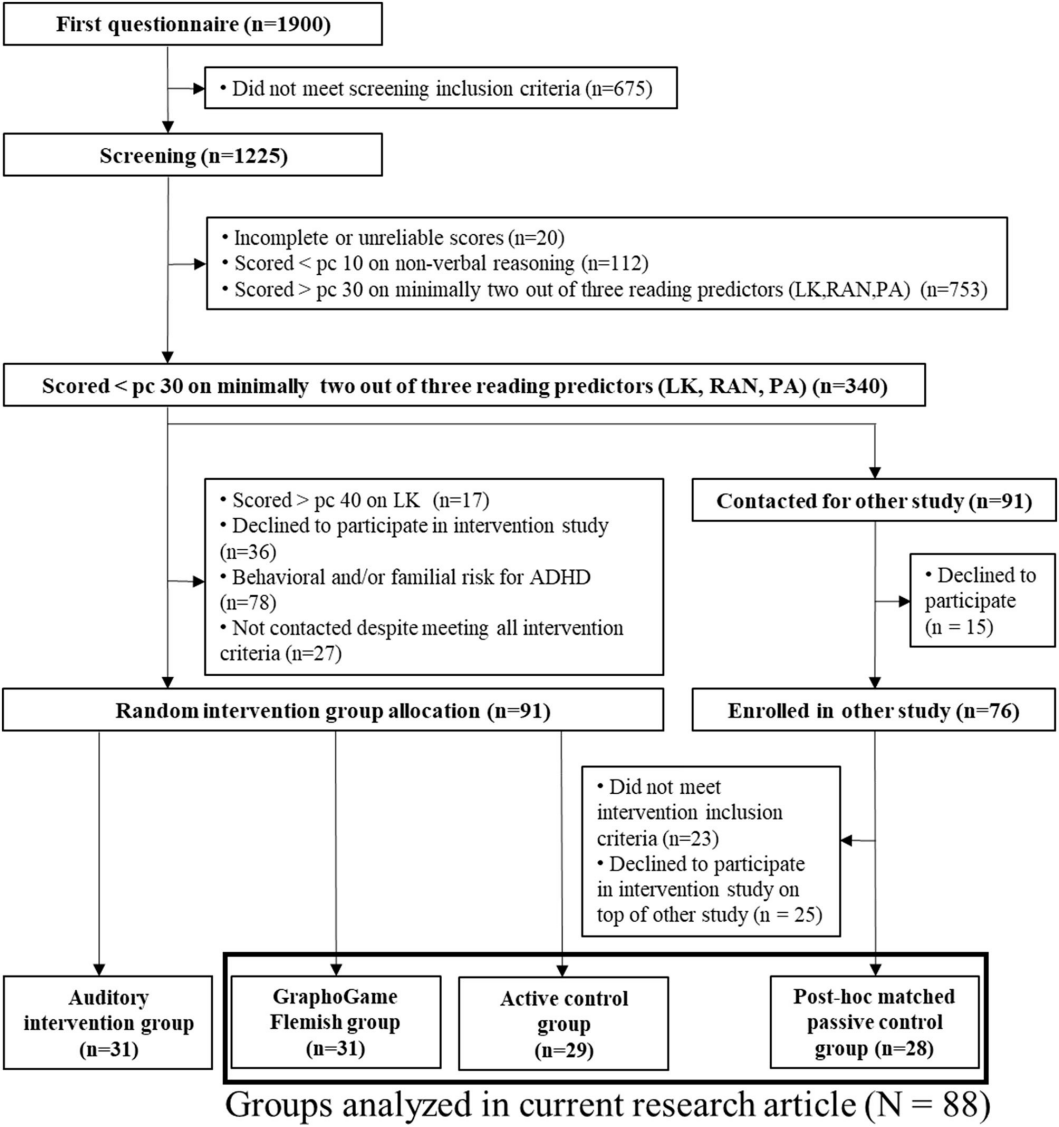
Flowchart of the participant selection from recruitment to group assignment.

In September 2018, a school-based screening took place at the start of the last kindergarten year, in which non-verbal reasoning skills and robust cognitive predictors of reading (PA, RAN, and LK) (Catts et al., 2001; De Jong and Van Der Leij, 2003) were assessed in 1,225 4- to 5-year olds. For feasibility reasons and to avoid the impact of phonological skills in the intelligence measure, only a non-verbal reasoning task was assessed as an approximation of general intellectual ability. A child was excluded in advance to take part in the screening when parental questionnaires indicated (1) therapy for language and/or articulatory problems, (2) a birth year other than 2013, (3) a history of severe hearing impairment, (4) neurological problems, (5) bilingualism, (6) a mother tongue other than Dutch, or (7) a total schooling period of <20 months. For a detailed description of the screening tasks and procedure, see Verwimp et al. (2020). Based on the screening test outcomes, parental questionnaires, and the Dutch Child Behavior Questionnaire indicating a potential behavioral risk for ADHD (Smidts and Oosterlaan, 2005), 91 Dutch-speaking children with an elevated cognitive risk for dyslexia, but without a behavioral and/or familial risk for ADHD enrolled in the intervention study. A cognitive risk for dyslexia was assigned when a child scored (1) below the 30th percentile on two out of the three reading-related measures (PA, LK or RAN) and (2) above the 10th percentile on non-verbal reasoning skills, i.e., a norm score above 75.3 on the Raven's Colored Progressive Matrices (Raven et al., 1984). In addition, since the experimental intervention largely focused on grapheme-phoneme coupling, children with both PA and RAN scores below the 30th percentile were only eligible for the intervention study when they also performed below percentile 40 on LK. According to Catts et al. (2001), the 30% cut-off scores are justified to estimate the risk for developing reading difficulties as they result in an optimal trade-off between specificity and sensitivity. Despite the chance of obtaining a high false-positive rate however, we still applied this cut-off as we were convinced that GG-FL could be beneficial also for children who would eventually not receive a formal diagnosis but still perform below-average on precursors of and/or actual reading tasks. By eliminating the lowest 10% scores of the non-verbal reasoning task, we ensured that only children within the normal range of intellectual ability could be included in the intervention study. More specifically, a cognitive functioning test with an average score of 100 and a standard deviation of 15, a score of two standard deviations below the average (with an error rate of five points) is considered as an indicator of intellectual impairment (i.e., a score between 65–75) (American Psychiatric Association, 2014). Based on a block randomization procedure (Coppock, 2019), the 91 at-risk children were randomly assigned to one of three experimental groups: (1) a GraphoGame Flemish group (*n* = 31) (GG-FL), (2) an active control group (*n* = 29) (AC), and an auditory intervention group (*n* = 31). It was ensured that the groups were matched in advance based on birth trimester, gender, and educational environment. Since disentangling the specific effects of the auditory intervention falls beyond the scope of the current study, the latter group will not be discussed any further. An extra group of 28 children served as the passive control group (PC) that received no intervention. They originally participated in another longitudinal dyslexia study, which recruited children from the same screening sample, but met the same inclusion criteria for the intervention study. Thus, the number of participants included in the current research article equals 88. Children from the PC group were not randomly assigned and were not a-priori matched to the children from the intervention groups. *Post-hoc* group comparisons, however, showed that the groups discussed in the current study did not differ regarding age, gender, SES, non-verbal IQ, home literacy environment (HLE) aspects, and maternal adult reading history questionnaire (ARHQ) scores, with higher scores indicating a lower maternal reading level (Lefly and Pennington, 2000) (see Table 1). There were significant group differences regarding paternal ARHQ scores. However, it must be noted that only 83 mothers and 71 fathers of the current study filled out the ARHQ questionnaire. HLE information, obtained through screening questionnaires, was also only available for 82 participants. For a more detailed overview of the participant recruitment procedure from screening to group allocation, see Figure 1. The study was approved by the Medical Ethical Committee of the University Hospital of Leuven, KU Leuven, and signed ethical consents were obtained for all participants.

**Table 1:** Demographic characteristics of the participants.

	**GG-FL**				**AC**				**PC**				**Statistic*_**(*df*)**_***	***p***
	***n***	***M***	***SD***	***Med***	***n***	***M***	***SD***	***Med***	***n***	***M***	***SD***	***Med***		
Age in months at pre-test	31	65.5	3.4	65.0	29	65.8	3.4	65.0	28	66.9	3.3	67.0	*H*_(2)_ = 3.37	0.186^b^
Non-verbal IQ^a^	31	101.2	16.9	101.9	29	96.3	13.4	95.5	28	94.9	14.3	93.4	*F*_(2, 85)_ = 1.43	0.246^c^
HLE (frequency of joint literate activities)^a^	29	−0.19	0.90	−0.17	25	−0.25	1.12	−0.27	28	0.30	0.83	0.29	*F*_(2, 79)_ = 2.75	0.070^c^
HLE (number of books at home)^e^	29	0.26	1.07	0.68	25	0.12	1.16	0.64	28	0.05	0.83	0.19	*H*_(2)_ = 2.73	0.255^b^
HLE (duration and frequency of reading)^e^	29	−0.18	0.82	−0.49	25	0.13	1.04	−0.50	28	0.09	0.97	−0.39	*H*_(2)_ = 3.75	0.153^b^
HLE (duration of joint literate activities)^e^	29	−0.16	0.96	−0.76	25	0.13	1.06	−0.67	28	0.25	1.03	0.26	*H*_(2)_ = 4.25	0.119^b^
Maternal ARHQ score^e^	30	0.06	0.86	−0.13	25	0.03	0.70	−0.10	28	−0.14	0.61	−0.10	*H*_(2)_ = 0.37	0.833^b^
Paternal ARHQ score^e^	21	0.33	1.29	0.27	22	0.57	0.95	0.76	28	−0.24	0.49	−0.08	*H*_(2)_ = 12.32	0.002^b^^*^
Gender (female/male)	31	16/15			29	13/16			28	17/11			*χ^2^*_(2)_ = 1.45	0.484^d^
SES (low/middle/high/unknown)	31	7/12/12/0			29	8/15/5/1			28	7/14/7/0			*$\chi_{\left(4\right)}^{2}$* = 3.39	0.495^d^

*GG-FL, GraphoGame Flemish group; AC, active control group, PC, passive control group; df, degrees of freedom; IQ, intelligence quotient; HLE, home literacy environment; ARHQ, adult reading history questionnaire; SES, socio-economic status; n, number of participants; M, mean; SD, standard deviation; Med, median. For categorical variables (gender and SES), the number of children in each subcategory is reported*.

a *Standardized scores (M = 100, SD = 15)*.

b *Independent-Samples Kruskal Wallis Test*.

c *One-way Anova test*.

d *Pearson Chi-Square test*.

e *Standardized factor scores based on parental screening questionnaires*.

* *p < 0.050*.

### Procedure

In the second half of the last year of kindergarten (January 2019), several specifically trained decoding-related skills (word decoding, productive and receptive LK, and PA), non-specifically trained phonological abilities (RAN and VSTM), and broader language skills (receptive vocabulary and morphological awareness) were evaluated. All assessments, except for those of receptive LK and PA, were individually administered at school in a quiet test room before and after the intervention period. The order of the tests was the same for all children. Immediately after pre-test measurements at school, all participants received a tablet (Samsung Galaxy Tab E9.6) with a headphone (Audiotechnica ATH M20x) and a manual for parents. The GG-FL and AC group were asked to play either a tablet-based version of GG-FL or an active control game respectively for 15 min per day, six days per week over a period of 12 weeks. This corresponded to an exposure time of 18 h/1,080 min spread over 84 days. Within the same period, both intervention groups also received an identical auditory tablet-based intervention, which comprised the active listening to recorded children stories for 10 min per day. This auditory intervention was almost identical to the auditory intervention of the abovementioned experimental group that was left out in the current research article. The only difference was that in this excluded group, the speech signals of the story recordings were adjusted to improve basic auditory speech perception. As such, comparing this group with the GG-FL and AC group would allow us to investigate a boosting effect on reading and phonology via enhanced speech perception (consider Van Herck et al., 2021 for further details of the auditory intervention and Van Hirtum et al., 2019, 2021 for its theoretical foundation). Disentangling this boosting effect however falls beyond the scope of the current study. Since the auditory intervention was identical in both the GG-FL as well as in the AC group simply to control for tablet exposure time, the design of the current study does not allow us to draw scientifically correct conclusions about its specific effects. All children, including the passive control group, were also asked to play two tablet-based mini-games at home assessing PA and receptive LK, every three weeks, for a total of five times, starting from the day after the pre-test measurement at school. In the manual, it was clearly emphasized that parents should not help their child during the game-based assessments and intervention games. After the intervention period, children in the experimental groups (GG-FL and AC) and their parents received a questionnaire regarding motivation and evaluation of the intervention.

### Assessments

#### Pre- and Post-Assessments: Specifically Trained Decoding-Related Skills

##### Word Decoding

Word decoding skills were assessed using an adapted version of the first reading card of the Drie-Minuten-Toets (DMT—version A, English: Three-Minute-Test) (Verhoeven, 1995). The 150 monosyllabic consonant-vowel, vowel-consonant, and consonant-vowel-consonant-words of the first reading card were printed onto 150 separate flash cards. During one min, the experimenter presented flashcard words and the child was asked to read them aloud. As children in Flanders do not receive any formal reading instruction in kindergarten (http://www.onderwijsvlaanderen.be/), the next word was presented when a child did not respond within 10 seconds. The test was interrupted when the child was not able to read five consecutive words correctly. Reading fluency was calculated based on the amount of words the child was able to decode correctly in one min.

##### Productive Letter Knowledge

Productive letter knowledge was assessed using a letter card with the 16 most frequently used letters in Dutch books (Boets et al., 2010). The child had to name each letter one by one. Both letter sounds and names were scored as correct. No feedback was given and the maximum score of the test was 16.

#### Intermediate Assessments: Specifically Trained Decoding-Related Skills

##### Phonological Awareness

Phonological awareness was measured through an end-phoneme identification task. This tablet-based task was embedded in a game-based assessment tool, called Diesel-X (Geurts et al., 2015) and was originally developed to define an early risk for dyslexia in pre-reading Dutch-speaking kindergarteners. In each trial, four objects were visually and binaurally presented on the tablet screen and through a headphone respectively. The child had to select the object with the same end-sound as a given target word. The task started with two training items for which feedback was given, followed by 10 test items without feedback. If necessary, children were allowed to replay the audio signal of the spoken test trials. There was no response time limit. The maximum score was 10.

##### Receptive Letter Knowledge

Receptive letter knowledge was also assessed within Diesel-X (Geurts et al., 2015). In this task, the letter sounds of the 16 most frequently used letters in Dutch books were binaurally presented to the child. The child had to indicate the correct corresponding letter symbol on the tablet screen out of five alternatives. No feedback was given, there was no response time limit, and the maximum score was 16.

#### Pre- and Post-Assessments: Non-Specifically Trained Phonological Abilities

##### Rapid Automatized Naming (RAN)

RAN of colors and objects (van den Bos et al., 2002) was assessed by presenting two cards with 50 symbols of five repetitive randomly ordered colors (black, yellow, blue, red, and green) and objects (tree, chair, duck, scissors, and bicycle), respectively. The child was required to name the colors and pictures (all monosyllabic words in Dutch) as accurately and fast as possible. The time needed to finish each card as well as the number of correctly named items were recorded. Since the error numbers were negligible across pre- and post-test and for both color and object naming, the final RAN score was an average score of the number of items (colors and objects) that a child could name correctly per second (the median of the total error amount was 0 for color and object naming, both in pre- and post-test. Interquartile range was 1 in color naming at pre-test and 0 for object naming in pre-test and both object and color naming at post-test).

##### Verbal Short Term Memory (VSTM)

VSTM was administered by means of a shortened version of the non-word repetition task (NRT) (De Smedt et al., 2009), which had been formerly used in a study with 5-year old kindergarteners with a family risk for dyslexia (Boets et al., 2010). The reason for choosing a non-word repetition task over forward or backward digit span tasks, also sometimes used to assess VSTM, was driven by the evidence for a reciprocal relationship between non-word repetition and reading development and by the absence of this reciprocal relationship regarding digit span tasks (Nation and Hulme, 2011; Cunningham et al., 2021). The non-word repetition task was administered on a computer which was connected with an external sound card (RME Fireface UC interface) via the software tool APEX (Francart et al., 2008). Non-words, which were in line with the phonotactic rules of Dutch, were monaurally presented through a calibrated HDA-200 headphone. The child was required to repeat them as accurately as possible. The test consisted of two training items in which feedback was given and 24 test items without feedback. The syllable length of the words in the test trials increased after every six items, ranging from two to five syllables. The maximum score of this test was 24.

#### Pre- and Post-Assessments: Non-Specifically Trained Broader Language Skills

##### Receptive Vocabulary

The Peabody Picture Vocabulary Test-III-NL (Dunn and Dunn, 2005) was used to evaluate receptive vocabulary. The test consisted of 17 subsets containing 12 test trials each. In each test trial, four pictures were presented and the child had to select the picture representing a given target word. The raw receptive vocabulary score was calculated by subtracting the sum of errors across all subtests from the number of the last item of the subset that was assessed last.

##### Morphological Awareness

Morphological awareness was assessed by means of the “Word Structure” subtest from the CELF-Preschool 2-NL (Wiig et al., 2012). This task measured the ability to apply morphological rules for conjugation, flexion, derivation, the degrees of comparison and rules for pronouns and cases. Each test item contained two pictures. The experimenter described the first picture (e.g., “This bird eats” / “Deze vogel eet”) and introduced the second picture with an incomplete sentence (e.g., “and this bird…” / “en deze vogel…”) in order to induce a response, in which the child had to apply a certain morphological rule of Dutch (e.g., “flies” — “vliegt” — morphological rule: conjugation of a Dutch regular verb in third singular form). As described in the official test manual, the test was interrupted when a child made more than seven consecutive errors. The maximum raw score was 23.

#### Questionnaires After the Intervention Period

After the intervention period, children from the experimental groups pointed out on a 5-point Likert scale ranging from 1 (“I did not like it at all”) to 5 (“I liked it very much”) how much they had appreciated the intervention games. Moreover, for the majority of the intervention children (GG-FL: *n* = 29, AC: *n* = 26), parental questionnaire information was obtained regarding (1) motivation of their child during the intervention period, (2) the time of the day when the intervention was usually played, (3) the amount of extra tablet exposure (in minutes per week) on top of the intervention games, (4) the encouragement needed to play, and (5) the frequency of shared story reading sessions per week during the intervention period. For those same 55 intervention children and all 28 passive control participants, extra information was obtained on (1) whether or not children received extra explicit reading instruction during the intervention period and (2) the amount and type of help that was offered during the Diesel-X sessions.

### Interventions

#### GraphoGame Flemish (GG-FL)

**Figure 2:**
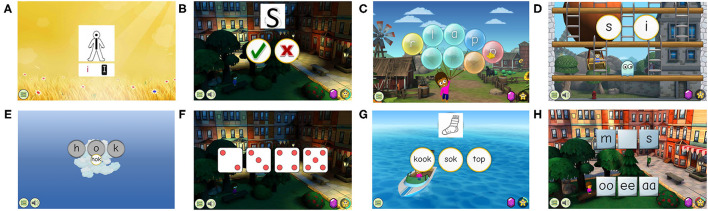
Examples of the different types of reading-related mini-games in GraphoGame Flemish. **(A)**, Grapheme story. **(B)**, Auditory discrimination. **(C)**, Visual discrimination. **(D)**, Grapheme-phoneme coupling. **(E)**, Phoneme blending. **(F)**, Phoneme counting. **(G)**, Reading. **(H)**, Spelling.

The GG-FL group received a Flemish version of GraphoGame (GG-FL) on a tablet, which was created based on the existing version from the Netherlands (Glatz, 2018). They were asked to play GG-FL six days per week, 15 min per day, over a period of 12 weeks. The classic GG content was extended based on the developmental reading model of Struiksma et al. (2004) in order to make the game more suitable for pre-reading children at risk. To specify, as opposed to the Dutch version from the Netherlands (GG-NL), which provided abrupt grapheme-phoneme mapping from the start, the learning content of GG-FL built up slowly by providing grapheme introduction stories, and auditory and visual discrimination exercises prior to grapheme-phoneme and reading training. GG-FL provided a total of 559 mini-games covering reading-related skills and 13 mini-games assessing playing motivation. There were eight different types of reading-related mini-games: grapheme introduction stories (*N* = 32) (Figure 2A), auditory discrimination (*N* = 80) (Figure 2B), visual discrimination (*N* = 198) (Figure 2C), grapheme-phoneme coupling (*N* = 107) (Figure 2D), phoneme blending (*N* = 53) (Figure 2E), phoneme counting (*N* = 25) (Figure 2F), reading (*N* = 30) (Figure 2G), and spelling (*N* = 34) (Figure 2H). For a more detailed description of the GG-FL build-up and game mechanics, see Glatz et al. (2021).

#### Active Control Intervention (AC)

The AC group was asked to play a tablet-based control game, also for 15 min per day, six days per week, over a period of 12 weeks. The control game consisted of six age-appropriate commercially available applications (Lego City My City, LegoDuploTown, LegoDuploTrains, Playmobil Horseriding, Playmobil Police, and Lego Heartlake Rush). Children were able to choose which games they played on each day, as long as they stuck to the advised playing duration.

#### Training Integrity

Although parents received a manual explaining the interventional procedure in detail, the research group was responsible for training fidelity and precisely followed up interventional engagement in all participants. Exposure times (in minutes) of all GG-FL and active control game sessions were logged on a University server and daily sent to the research group, so that parents of children who did not play according to the advised session durations were contacted and encouraged to increase playing times.

### Statistical Analysis

All analyses were performed in IBM SPSS Statistics version 27 (IBM Corp., 2020) and data visualization was done in RStudio (RStudio Team, 2019). First, groups were compared on pre-test assessments and intervention-related factors that could influence the interpretation of possible intervention effects through either Mann-Whitney U, One-way ANOVA, or Fisher's Exact tests, depending on the nature and distribution of the dependent variable of interest. Intervention-related factors included intervention exposure (GG-FL/AC, story intervention, and total combined exposure), total training duration in days to complete both interventions, extra tablet exposure in minutes on top of the interventional games, motivation reported by the parent and child, usual daily playing moment, and the amount of story reading sessions per week. Story intervention data of four children in the GG-FL group were lost due to technical tablet problems. For that reason, the story intervention exposure, the total combined tablet exposure, and the total training duration to complete both interventions could only be calculated for 27 children in the GG-FL group. The second analysis step involved the examination of intervention effects. After checking assumptions and fit indices, linear growth differences between groups regarding productive and receptive LK, PA, RAN, VSTM, receptive vocabulary, and morphological awareness were examined using linear mixed models (LMMs). Considering the LLMs for variables with only two assessment points, group and time were specified as a between-subjects factor and a dummy-coded predictor (0: pre-test, 1: post-test) respectively. Intercepts were allowed to vary across subjects. As for the variables with multiple assessment points in the Diesel-X tasks (e.g., receptive LK and PA), LMMs with an unstructured covariance pattern and randomly varying intercepts and slopes across subjects were specified with group as a between-subjects factor and time as a continuous predictor in terms of training days. Data from a Diesel-X task, either a receptive LK- or PA-task, were excluded from the dataset when the assessment was incomplete. Furthermore, data of all Diesel-X trials from a child were excluded when parents reported that their child received content-related support during any of the Diesel-X sessions. This was the case for six participants. Although we demanded to only play five Diesel-X sessions, a minority of children played six or seven times. Given that LMMs deal with missing values and time was specified as continuous in terms of training days, data of these extra Diesel-X sessions were kept in the dataset (see Table 2 for final group distributions across Diesel-X session trials). Given the violated assumptions to perform LMMs in word decoding, differences in progress between groups were analyzed using generalized estimation equations (GEE). Based on the right-skewed distribution and the predominant amount of zero-scores in word decoding (i.e., floor effects), a Tweedie distribution model was specified with group and time as a between-subjects factor and a dummy-coded predictor (0: pre-test, 1: post-test) respectively. In all analyses, the AC group was set as the reference category and the group^*^time interaction was considered as an indication of growth differences between groups. In case of a significant interaction, the slope parameters of the model were considered to compare the slopes of the GG-FL and the PC group against the slope of the reference AC group. As such, we could (1) explore the direct impact of GG-FL against AC and rule out any potential placebo effects of general tablet playing and (2) double-check whether the AC games did also not accidentally train the dependent reading-related variable of interest.

**Table 2:** Group distributions across Diesel-X session trials.

	**Number of participants**					
	**Receptive LK task**			**PA task**		
	**GG-FL**	**AC**	**PC**	**GG-FL**	**AC**	**PC**
First trial (T1)	29	25	26	27	25	26
Second trial (T2)	28	24	26	28	23	26
Third trial (T3)	27	23	26	27	22	25
Fourth trial (T4)	25	23	25	24	22	25
Fifth trial (T5)	22	18	21	21	17	21
Sixth trial (T6)	5	5	4	5	5	4
Seventh trial (T7)	1	0	0	1	0	0

*LK, letter knowledge; PA, phonological awareness; GG-FL, GraphoGame Flemish group; AC, active control group; PC, passive control group*.

## Results

The result section is divided into different sections describing (1) the group comparisons of baseline measures at pre-test and intervention-related factors, (2) intervention-driven effects on specifically trained decoding-related skills (word decoding, productive LK, receptive LK, and phonological awareness), (3) effects on non-specifically trained phonological abilities (RAN and VSTM), and (4) on non-specifically trained broader language skills (receptive vocabulary and morphological awareness).

### Baseline Measures at Pre-Test and Intervention-Related Factors

**Figure 3:**
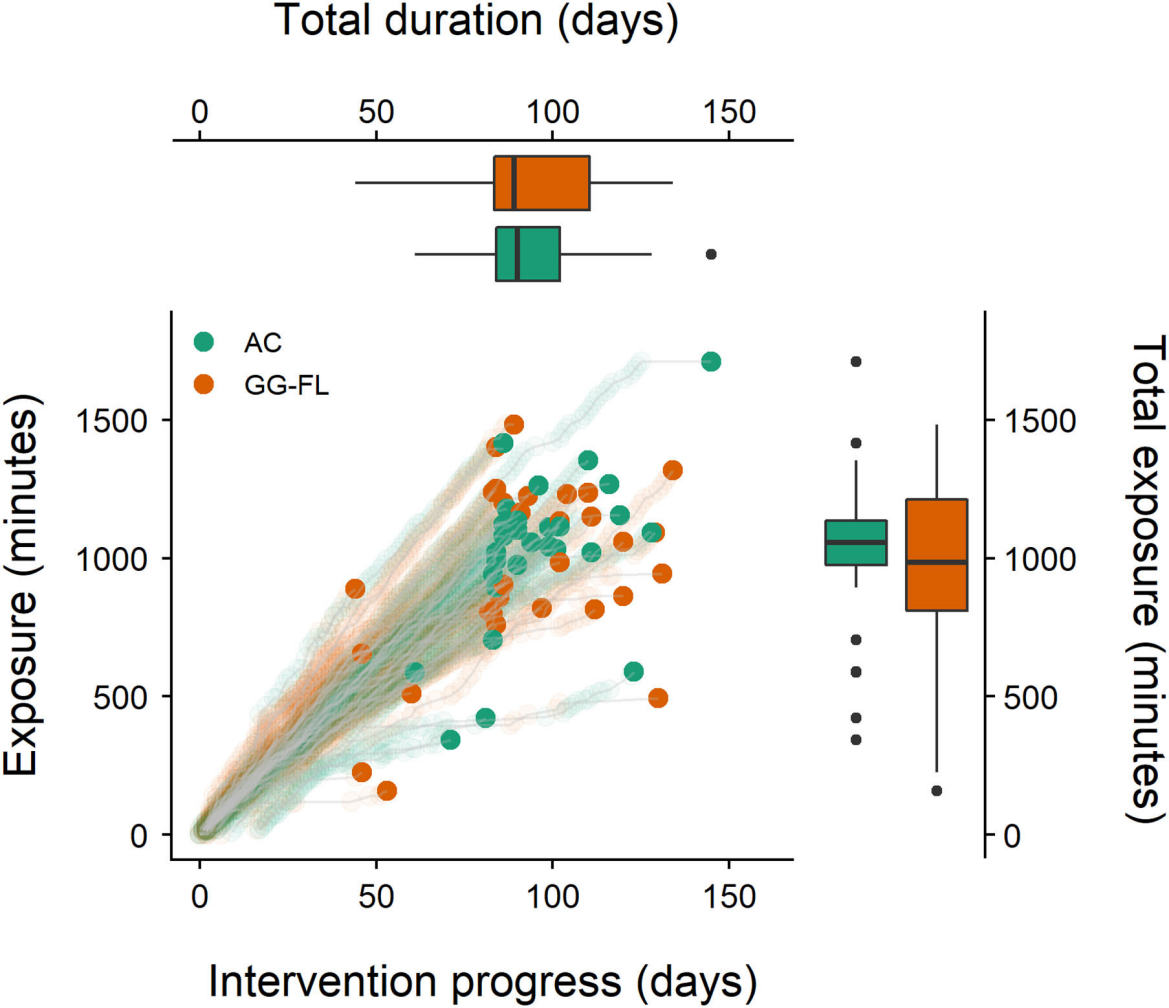
Individual interventional AC/GG-FL trajectories and total training GG-FL/AC duration and exposure. AC, active control group; GG-FL, GraphoGame Flemish group. The bold dots represent an individual's last GG-FL/AC playing day and highest obtained GG-FL/AC intervention exposure. Boxplots represent the overall group distributions of total GG-FL/AC exposure and total GG-FL/AC training duration.

At the start of the intervention, groups were comparable concerning the specifically trained decoding-related skills (active LK: *H*_(2)_ = 0.37, *p* = 0.831, word decoding: *H*_(2)_ = 0.40, *p* = 0.820, receptive LK: *H*_(2)_ = 0.42, *p* = 0.809, PA: *H*_(2)_ = 0.14, *p* = 0.933), the non-specifically trained phonological abilities (RAN: *F*_(2, 85)_ = 0.11, *p* = 0.898, VSTM: *F*_(2, 85) =_ 1.10, *p* = 0.339), and the broader language skills (morphological awareness: *F*_(2, 85)_ = 0.47, *p* = 0.626, receptive vocabulary: *H*_(2)_ = 2.54, *p* = 0.280). In addition, we were certain that our participants were pre-reading at pre-test, as none of the children across all three groups were able to decode more than one word correctly. None of the parents reported any form of explicit reading instruction during the intervention period. Table 3 also shows no group differences on any of the intervention-related factors. Overall, although intervention groups played a comparable amount of GG-FL/AC game, the general exposure times were more or less one/two h lower than the demanded playing exposure of 18 h. Moreover, the overall training period to finish both the GG-FL/AC and story intervention was ~12–13 days longer than the expected 84 days in both groups. However, Figure 3, which presents an overview of the individual interventional GG-FL/AC trajectories combined with the distribution of the total GG-FL/AC training duration and exposure time across groups, clearly shows that the majority of the participants played ~1,000 min within 84–105 days. These results indicate that training integrity was acceptable.

**Table 3:** Group comparisons of intervention-related factors.

	**GG-FL group**					**AC group**					***statistic***	***p***
	***n***	***M***	***SD***	***Med***	***IQR***	***n***	***M***	***SD***	***Med***	***IQR***		
GG-FL/AC exposure (hours)	31	16.01	5.32	16.40	6.97	29	17.09	4.75	17.59	3.08	*U =* 411.00	0.569^a^
Story intervention exposure (hours)	27	11.15	2.77	12.10	0.35	29	11.38	1.90	12.10	0.20	*U* = 378.50	0.797^a^
Total intervention exposure (hours)	27	27.19	7.45	28.77	6.97	29	28.47	6.28	29.29	2.99	*U* = 361.00	0.617^a^
Motivation reported by child (Likert scale)	31	3.07	1.57	3.00	3.00	29	3.14	0.53	3.00	0.70	*U* = 416.50	0.623^a^
Extra tablet exposure (minutes/week)	29	80.00	119.24	0.00	143.00	26	33.33	55.83	0.00	58.00	*U* = 246.50	0.345^a^
Overall training duration of GG-FL/AC and story intervention combined (days)	27	96.70	20.33	95.00	25.00	29	97.55	17.26	93.00	26.50	*F*_(1, 54)_ = 0.03	0.867^b^
Motivation reported by parent (VM/RM/ID/NM/unknown)	29	5/13/2/5/4				26	5/15/0/5/1				-	0.551^c^
Encouragement to play (ENN/ESN/EMN/EAN/unknown)	29	3/16/3/4/3				26	8/13/4/1/0				-	0.126^c^
Usual playing moment (MN/N/A/E/V/unknown)	29	4/0/3/14/7/1				26	4/1/4/13/4/0				-	0.879^c^
Book reading sessions(never/rarely/once a week/ multiple times per week/daily/unknown)	29	9/1/2/10/6/1				26	2/3/2/9/10/0				-	0.168^c^

*GG-FL, GraphoGame Flemish; AC, active control game; n, number of participants; M, mean; SD, standard deviation; Med, median; IQR, interquartile range. For categorical variables (motivation reported by parent, encouragement to play, usual playing moment, and book reading sessions), the number of children in each subcategory is reported; VM, very motivated; RM, relatively motivated; ID, independent; NM, not motivated; ENN, encouragement never needed; ESN, encouragement sometimes needed; EMN, encouragement mostly needed; EAN, encouragement always needed; MN, morning; N, noon; A, afternoon; E, evening; V, varying playing moment*.

a *Mann-Whitney U test*.

b *One-way ANOVA test*.

c *Fisher's Exact test*.

### Specifically Trained Decoding-Related Skills

#### Pre- and Post-Test Assessments

**Figure 4:**
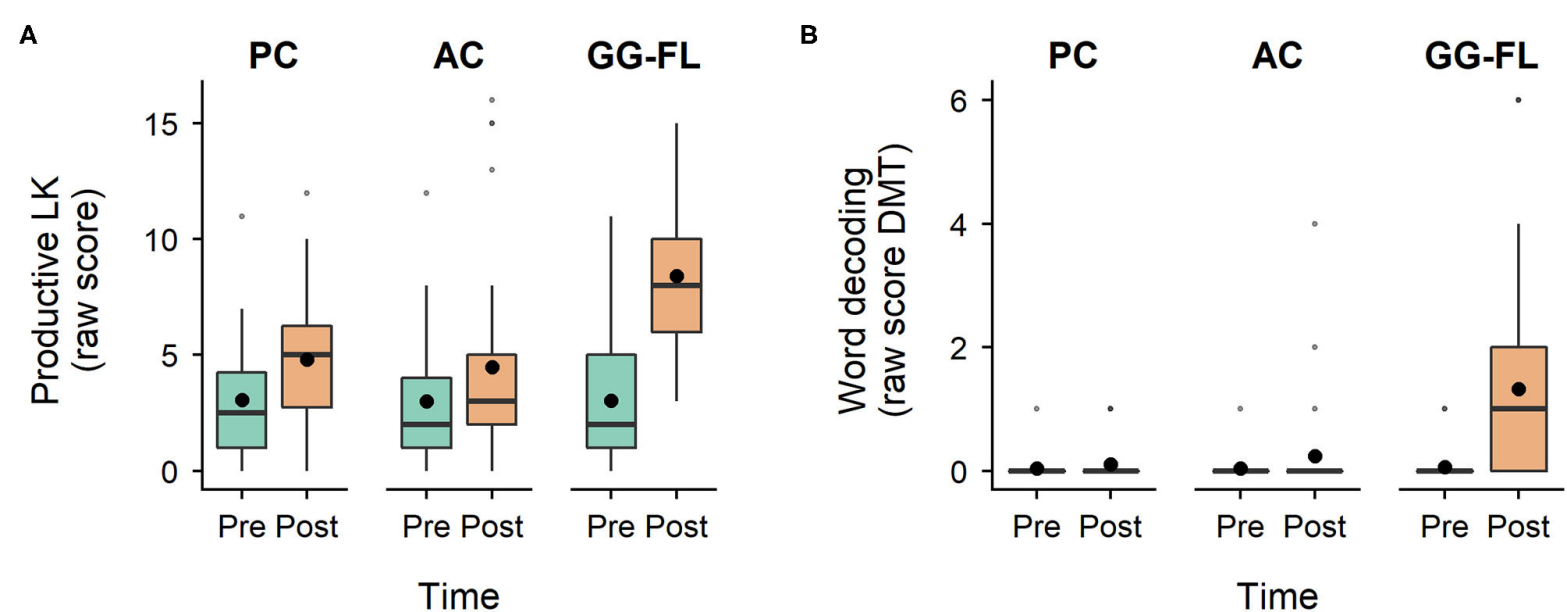
Specifically trained decoding-related skills—pre- and post-test assessments. **(A)**, Distribution of productive letter knowledge scores across groups and timepoints, **(B)**, Distribution of word decoding scores across groups and timepoints. LK, letter knowledge; DMT, (Drie-Minuten-Toets, English: Three-Minute Test); PC, passive control group; AC, active control group; GG-FL, GraphoGame Flemish group. Black dots represent the mean of the raw scores.

Figure 4A shows the distribution of raw productive LK data across groups and timepoints. The LMM revealed a main effect of time (*F*_(1, 85)_ = 133.47, *p* < 0.001), no main effect of group (*F*_(2, 109.73)_ = 0.003, *p* = 0.997), and a significant group^*^time interaction (*F*_(2, 85)_ = 26.40, *p* < 0.001). A significant progress in the reference AC group from pre- to post-test was found (β = 1.48, *SE*_β_ = 0.43, *t*_(85)_ = 3.43, *p* = 0.001, 95% CI [0.62, 2.34]). Moreover, the model revealed a significantly steeper progress in productive LK for the GG-FL compared to the reference AC group (β = 3.90, *SE*_β_ = 0.60, *t*_(85)_ = 6.48, *p* < 0.001, 95% CI [2.70, 5.10]), whereas no growth differences between the AC and PC group were established (β = 0.27, *SE*_β_ = 0.62, *t*_(85)_ = 0.43, *p* = 0.666, 95% CI [-0.96, 1.50]). As for word decoding, presented in Figure 4B, the GEE analysis revealed a main effect of time (Wald χ^2^_(1)_ = 19.68, *p* < 0.001), no main effect of group (Wald χ^2^_(2)_ = 0.34, *p* = 0.843), and a significant group^*^time interaction (Wald χ^2^_(2)_ = 13.76, *p* = 0.001). Model estimates revealed no significant progress from pre- to post-test in the reference AC group (β = 0.21, *SE*_β_ = 0.12, Wald χ^2^_(1)_ = 2.82, *p* = 0.093, 95% Wald CI [−0.03, 0.45]) and no growth differences between this reference group and the PC group (β = −0.14, *SE*_β_ = 0.14, Wald χ^2^_(1)_ = 0.91, *p* = 0.339, 95% Wald CI [−0.41, 0.14]). Yet, the GG-FL group made significantly more progress compared to the reference AC group (β = 1.05, *SE*_β_ = 0.34, Wald χ^2^_(1)_ = 9.61, *p* = 0.002, 95% Wald CI [0.39, 1.72]). These results indicate (1) no accidental training effect of the AC game, and (2) a positive training effect of GG-FL on both productive LK and word decoding.

#### Intermediate Assessments

**Figure 5:**
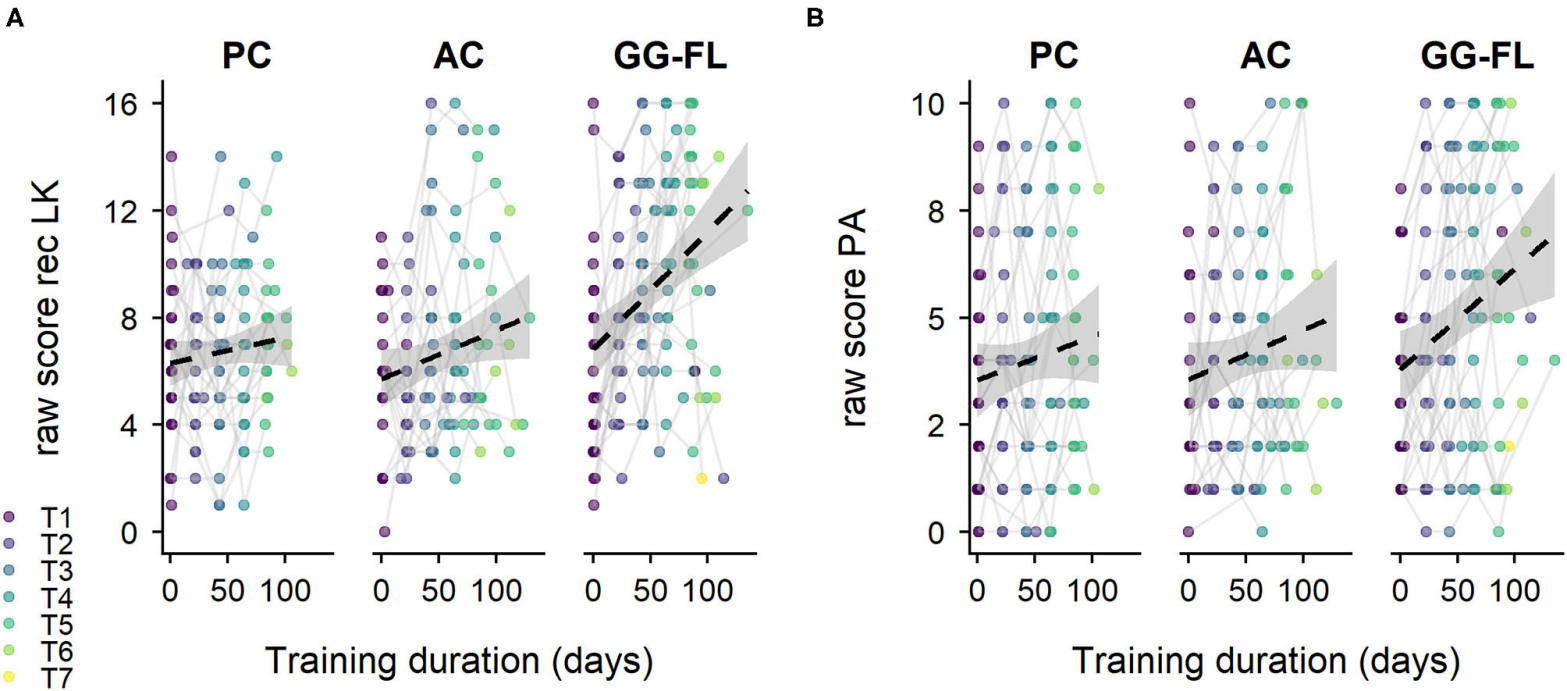
Specifically trained decoding-related skills—intermediate assessments. **(A)**, Raw receptive letter knowledge scores. **(B)**, Raw phonological awareness scores. rec LK, receptive letter knowledge; PA, phonological awareness; PC, passive control group; AC, active control group; GG-FL, GraphoGame Flemish group; T1-T7, trial 1–trial 7. The dashed lines represent the estimated linear slope.

Receptive LK and phonological awareness scores across groups and training days are shown in Figures 5A,B respectively. With regard to receptive LK, the LMM showed a main effect of time (*F*_(1, 70.79)_ = 31.47, *p* < 0.001), no main effect of group (*F*_(2, 73.59)_ = 0.43, *p* = 0.652), but a significant group^*^time interaction (*F*_(2, 70.65)_ = 6.95, *p* = 0.002). The model estimates showed a significant receptive LK progress from pre- to post-test in the AC group (β = 0.02, *SE*_β_ = 0.01, *t*_(64.49)_ = 2.16, *p* = 0_._034, 95% CI [0.001, 0.036]). Moreover, the GG-FL group learned quicker than the AC group (β = 0.03, *SE*_β_ = 0.01, *t*_(66.36)_ = 2.85, *p* = 0_._006, 95% CI [0.01, 0.06]), whereas the slopes of the AC and PC groups were comparable (β = −0.01, *SE*_β_ = 0.01, *t*_(71.74)_ = −0.59, *p* = 0.555, 95% CI [−0.03, 0.02]). As for phonological awareness, a main effect of time (*F*_(1, 63.59)_ = 32.41, *p* < 0.001), no main effect of group (*F*_(2, 73.56)_ = 0.10, *p* = 0.903), and an insignificant group^*^time interaction (*F*_(2, 63.46)_ = 2.63, *p* = 0.080) was found. Model estimates showed a significant progress from pre- to post-test in the AC group (β = 0.01, *SE*_β_ = 0.01, *t*_(57.48)_ = 2.40, *p* = 0.019, 95% CI [0.002, 0.024]) and a comparable growth between the latter and the PC group (β = 0.00, *SE*_β_ = 0.01, *t*_(65.29)_ = −0.12, *p* = 0.904, 95% CI [−0.02, 0.02]). Yet, the growth difference between the GG-FL and AC group almost reached significance (β = 0.01, *SE*_β_ = 0.01, *t*_(58.55)_ = 1.89, *p* = 0.063, 95% CI [0.00, 0.03]).

### Non-Specifically Trained Phonological Abilities

**Figure 6:**
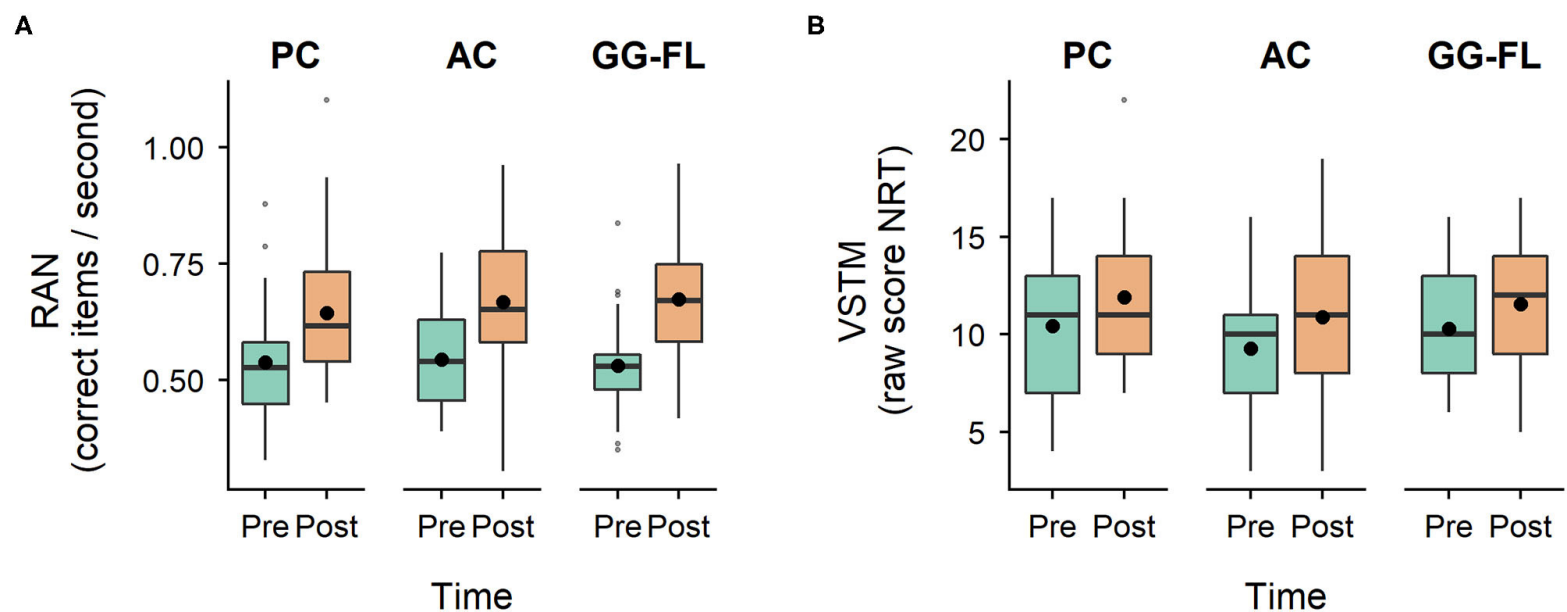
Non-specifically trained phonological abilities. **(A)**, Distribution of rapid automatized naming scores across groups and timepoints. **(B)**, Distribution of verbal short-term memory scores across groups and timepoints. RAN, rapid automatized naming; VSTM, verbal short-term memory; NRT, non-word repetition task; PC, passive control group; AC, active control group; GG-FL, GraphoGame Flemish group. Black dots represent the mean of the raw scores.

RAN and VSTM scores across groups and timepoints are presented in Figures 6A,B respectively. Considering RAN, we observed a significant main effect of time (*F*_(1, 85)_ = 118.81, *p* < 0.001), but neither a significant main effect of group (*F*_(2, 118.16)_ = 0.08, *p* = 0.921), nor a significant group^*^time interaction (*F*_(2, 85)_ = 0.86, *p* = 0.425). The same outcomes were found for verbal short-term memory: a main effect of time (*F*_(1, 85)_ = 20.07, *p*<*0*.001), but an insignificant main effect of group (*F*_(2, 122.51)_ = 0.95, *p* = 0.392) and group^*^time interaction (*F*_(2, 85)_ = 0.11, *p* = *0*.897).

### Non-Specifically Trained Broader Language Skills

**Figure 7:**
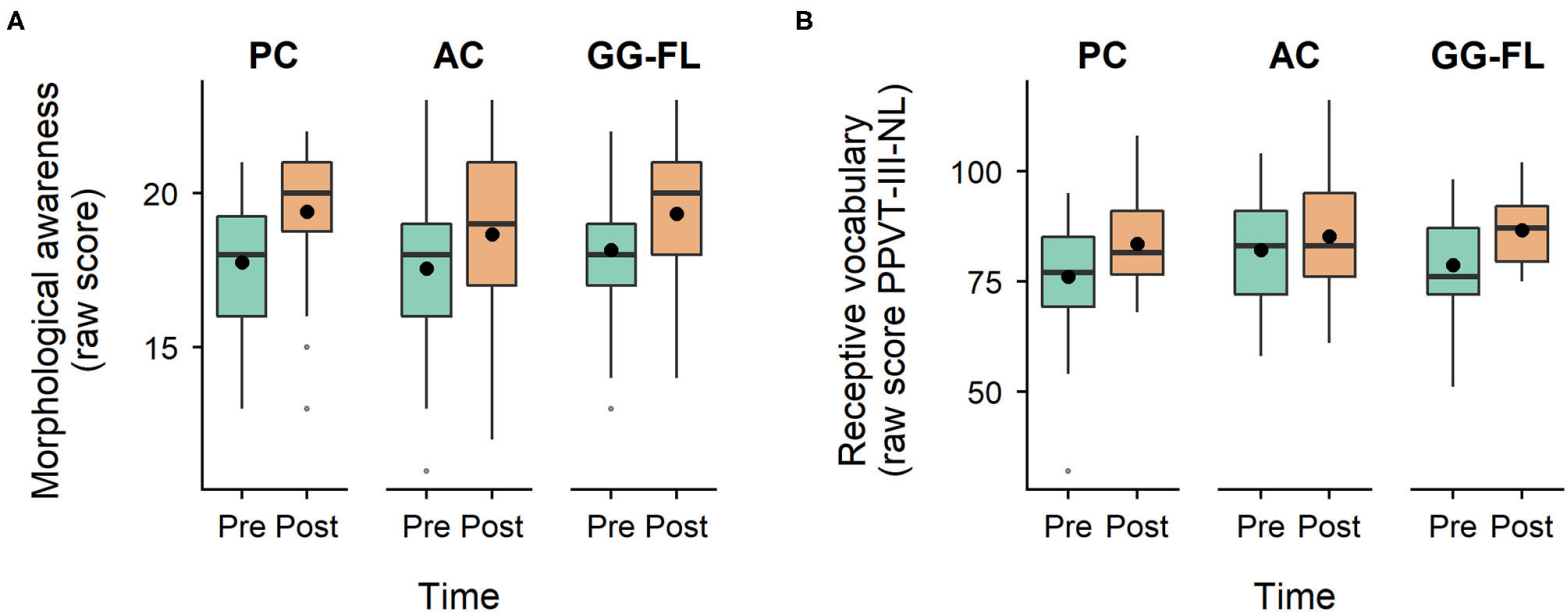
Non-specifically trained broader language skills. **(A)**, Distribution of morphological awareness scores across groups and timepoints. **(B)**, Distribution of receptive vocabulary scores across groups and timepoints. PPVT-III-NL, Peabody Picture Vocabulary Task-III-NL; PC, passive control group; AC, active control group; GG-FL, GraphoGame Flemish group. Black dots represent the mean of the raw scores.

Distributions of morphological awareness and receptive vocabulary scores across groups and timepoints are shown in Figures 7A,B respectively. Regarding morphological awareness, the LLM showed a significant main effect of time (*F*_(1, 85)_ = 24.87, *p* < 0.001), but an insignificant group (*F*_(2, 136.11)_ = 0.49, *p* = 0.615) and group^*^time effect (*F*_(2, 85)_ = 0.42, *p* = 0.660). The same results were obtained for receptive vocabulary. The analysis revealed a significant main effect of time (*F*_(1, 85)_ = 19.61, *p* < 0.001), but insignificant group (*F*_(2, 148.29)_ = 1.88, *p* = 0.157) and group^*^time effects (*F*_(2, 85)_ = 1.20, *p* = 0.306).

## Discussion

In the present study, we explored the impact of a preventive game-based GG-FL intervention on specifically trained decoding-related skills and non-specifically trained phonological and broader language skills in Dutch-speaking kindergarteners at cognitive risk for dyslexia. The intervention effects were evaluated by comparing changes in reading-related skills of at-risk children who played either GG-FL, an active control game, or no game at all. As hypothesized, while the growth of the AC and PC groups was comparable across time, the GG-FL group showed advantages on productive and receptive letter knowledge, and word decoding over the AC group. As for phonological awareness, results were less clear given the trend toward a growth difference between the GG-FL and AC group. As expected, based on previous studies, we established no GG-FL driven transfer effects on non-trained phonological abilities and broader language skills related to reading. Since the amount of exposure to GG-FL vs. the AC game and to the story intervention was comparable across intervention groups and groups did not differ on any of the intervention-related factors, the growth differences between groups concerning letter knowledge, word decoding, and phonological awareness in some extent, could be explained by the efficacy of GG-FL. Moreover, these results also assured that the AC game did not accidentally train any of the reading-related variables.

Our promising findings support prior preventive (Brem et al., 2010) and non-preventive GraphoGame research (McTigue et al., 2019), and other studies using real-life or digital game-based phonics interventions in kindergarten (Schneider et al., 2000; Elbro and Petersen, 2004; Regtvoort and Van Der Leij, 2007; Bowyer-Crane et al., 2008; Kegel et al., 2009; Van Otterloo and Van Der Leij, 2009; Kegel and Bus, 2012; Savage et al., 2013; Piquette et al., 2014). Moreover, although we did not directly compare the GG-FL approach with other approaches within the same study, our results correspond to the general advice to use a phonics-based approach when targeting decoding skills (National Institute of Child Health Human Development, 2000; Snowling and Hulme, 2011; Galuschka et al., 2014).

When comparing our results to those of Lovio et al. (2012), our clear LK and word decoding impact carefully raises the idea that (1) augmenting the training duration and (2) changing the general GraphoGame method by adding extra auditory and visual discrimination exercises, are necessary adjustments to stimulate reading development in pre-reading children at cognitive risk for dyslexia. Yet, cross-linguistic comparisons between intervention effects (Finnish vs. Dutch) are rather uncertain, as the developmental pattern of learning to read also relies on the schooling program (Kindall et al., 2018) and the orthographic depth of a language (Seymour et al., 2003; Georgiou et al., 2008). Thus, in order to rule out these additional influences and make evidence-based inferences about the impact of adjustments in reading intervention programs, it is of relevance to conduct randomized control trials using several versions of the same intervention program (with and without adjustments) in samples speaking the same language, and following the same educational trajectory. Although GG-FL was created based on the existing Dutch GraphoGame of the Netherlands (GG-NL) (Glatz, 2018), the latter could not be used for comparison in the current study due to (1) major differences in pronunciation between Northern Dutch (spoken in the Netherlands) and Southern Dutch (spoken in Flanders), (2) the fact that GG-NL was originally intended as a 6-week intervention for children in first grade who already received formal reading instruction, and (3) its former evaluation in entire classrooms and thus not only in children at risk for/with reading difficulties.

Another point of discussion involves the less clear impact of GG-FL on PA. Although the extra auditory and visual discrimination exercises were actually intended to train PA more compared to previous GraphoGame studies (Brem et al., 2010; Lovio et al., 2012; McTigue et al., 2019), our results did not reveal a clear growth difference between the AC and GG-FL group. A first valid explanation for this finding could involve the way PA was assessed. To clarify, GG-FL mainly targeted PA by means of auditory discrimination, and to a limited extent, phoneme counting and blending. Explicit end-phoneme identification tasks did not specifically belong to the intervention content. If GG-FL would have contained end-phoneme identification exercises or if we would have assessed phonemic awareness based on the way it was trained, we might have found a clearer growth difference between groups. Yet, this speculated training specificity raises the question whether the current PA exercises embedded in GG-FL are suitable to generate transfer effects on reading ability via trained phonological awareness later on. Although Struiksma et al. (2004) emphasized the importance of visual and auditory discrimination exercises in reading development, several meta-analyses advised letter-sound training combined with phoneme blending to obtain the most effective results (Snowling and Hulme, 2011; Galuschka et al., 2014). Increasing the amount blending exercises in GG-FL might result in clearer PA effects. A second explanation corresponds to the idea that PA is generally difficult to train in children with poor literacy skills (Hatcher et al., 1994), an assumption that could also explain the mixed findings of PA effects in previous intervention studies that combined a reading and phonological awareness training component (Elbro and Petersen, 2004; Bowyer-Crane et al., 2008; Van Otterloo and Van Der Leij, 2009; Savage et al., 2013; Piquette et al., 2014). A third explanation relates to the orthographic depth of Dutch, irrespective of the fact that the meta-analysis of McTigue et al. (2019) did not find a significant influence of orthographic depth on the efficacy of GraphoGame. Reading instruction seems to influence PA at a faster rate in transparent than opaque orthographies (Goswami et al., 2001). Dutch is considered as semi-transparent (i.e., not as transparent as Finnish or Italian, but not as opaque as English or Danish) (Seymour et al., 2003). Since our participants were pre-reading and at risk for reading failure based on low phonological skills, the GG-FL training, which specifically trained PA but mainly offered phonics-instruction, was possibly not sufficient enough (when played 15 min per day, 6 days per week over a period of 12 weeks) on its own to foster PA skills in a semi-transparent language, such as Dutch. Since Finnish is more transparent than Dutch, it could be possible that Lovio et al. (2012) might have revealed PA effects when their intervention duration and intensity was equal to the one used in the current study. Cross-linguistic research is needed to clarify this issue. A last alternative explanation for the less steep PA growth relates to the Diesel-X assessment procedure. Although research suggests that game-based assessments increase practical and economic feasibility (Cernich et al., 2007), decrease test anxiety (Kiili and Ketamo, 2018), and enhance motivation leading to more precise test outcomes (Bellotti et al., 2013; Geurts et al., 2015), it remains of high relevance, even in an automated assessment, to exercise supervision over the test environment (Moser et al., 2011; Bellotti et al., 2013; Vaughan et al., 2014). In all three groups and across all Diesel-X sessions, the lack of supervision in the home-based assessments might have led to distraction, misunderstanding of the test instructions, or social interactions that were not allowed during the assessments, e.g., receiving content-related support from parents/siblings (Vaughan et al., 2014). These types of uncontrolled error sources might have under- (in case of distraction or instruction misunderstanding) or overestimated (in case of support) the performance in some of the Diesel-X assessments (Moser et al., 2011). Possibly, this so-called data-noise still allowed for a clear receptive LK effect, which was also reflected in the productive LK outcome, but potentially masked a smaller PA effect. Figure 5 indeed shows that even at T1, which is considered as a baseline measure, a subset of children in all three groups almost obtained maximum scores in the Diesel-X assessment tasks. Given that the children were selected based on low PA, LK, and RAN outcomes, these observations lead us to suspect that some children indeed received any form of content-wise support from parents/siblings. This finding not only raises doubts on the reliability and quality of our home-based assessment outcomes, but also on the implementation of unsupervised home-based assessments in such a young age-group in general. Thus, the home-based uncontrolled assessment outcomes in the current study must be interpreted with caution. Fortunately, we still found positive intervention-driven effects in the more controlled individual school-based test assessment sessions.

Although we did not expect transfer effects on non-trained phonological and broader language skills based on the null-findings of previous studies, we could argue that the absence of an influence on VSTM and RAN was surprising to some extent. To clarify, the beginning stages of reading development in Dutch mainly involve the acquisition of the alphabetic principle (Verhoeven and Perfetti, 2017). Decoding stimulates the segmentation of whole word sounds into separate phonemes (Frith, 1998), thereby refining phonological representations. As such, GG-FL, which unraveled the principles of decoding, could have led to an improved ability to repeat novel phonological combinations, such as non-words (Nation and Hulme, 2011). Furthermore, learning to read increases the efficiency to convert visual items into phonological codes, a linguistic process which is also highly involved in RAN-tasks (Peterson et al., 2018). In the current study, it is plausible that reading performance was still too basic at post-test to yield transfer-effects on broader language, but even on untrained phonological skills that are more closely related to the decoding process of reading. Indeed, in accordance with the findings of Brem et al. (2010), reading performance also remained rudimental in the current study, since only three children in the GG-FL group were able to read more than five words at post-test. However, the GG-FL group might possibly benefit from the head start on LK and word decoding within and beyond the trained abilities, when they will receive explicit formal reading instruction from first grade onwards.

A last point of discussion concerns the general feasibility of the current home-based digital learning intervention. Although a large subset of children were exposed to the minimally demanded 1,080 min of GG-FL/AC, we still observed variation in final exposure time and training duration in terms of days. Moreover, the overall training exposure to finish both the GG-FL/AC and story intervention was generally less than demanded in both groups, a finding that was also reported in the home-based intervention study of Van Otterloo and Van Der Leij (2009). Furthermore, concerning the mandatory Diesel-X assessments every three weeks, Table 2 showed that even in the first trial, original group sizes were already reduced with a drop-out of ~one third of the children per group at the fifth trial. A subset of children thus simply never played Diesel-X or did not complete all five assessments. The variation in training days, intervention exposure, and the Diesel-X drop-out throughout the course of the intervention presumes that the intensity of GG-FL/AC (6 days per week, 15 min over 12 weeks) combined with an extra story intervention and regular Diesel-X assessments was maybe too much to ask for some children/parents. These findings cast doubts upon whether the home environment was the most optimal setting for this relatively intensive tablet-based reading intervention in such a young pre-reading age group. A previous GraphoGame study revealed that children who received a school-based intervention showed enhanced engagement and played more sessions compared to children who received GG at home (Ronimus and Lyytinen, 2015). Ronimus and Lyytinen (2015) also reported that teachers controlled playing times better than parents, raising the idea that school is a more structured environment to undergo an intervention program on a nearly daily basis. Yet, the variability in terms of training days, exposure, and intensity, but also variability in the individual in-game properties (overall game accuracy, overall responses given, amount of mini-games played, items seen on the tablet-screen) is of relevance for future studies to model under which conditions GG-FL can be played best to obtain the largest gains in early literacy skills.

We acknowledge two main limitations of the present study. First, given that our participants were still too young for standardized reading and spelling measurements at the end of the study, the real potential of GG-FL to actually overcome the development of reading difficulties still remains unclear. We plan to conduct a follow-up study in order to assess actual reading and writing based on standardized test materials. This scheduled follow-up study will also contain a group of typically-developing peers in order to investigate whether GG-FL is able to narrow or even close the achievement gap in reading and spelling abilities. A second limitation concerns the use of our home-based assessments of PA and receptive LK. Although intermediate test sessions allow for more precise parameter estimates and game-based assessments enjoy several benefits (O'Leary et al., 2018), the lack of controlled test supervision at home probably led to less reliable test outcomes of PA and receptive LK. Future studies who are planning to make use of digital home-based e-assessments are recommended to foresee an online supervisor in order to exercise control over the test environment and obtain reliable data.

In sum, our findings assume a GG-FL-driven head start in letter knowledge and word decoding in pre-readers at cognitive risk for dyslexia. These promising results thereby support the potential of preventive digital game-based and phonics-focused interventions in children who already show difficulties in key precursors of later reading ability. In the future, we hope to establish a long-term effect in order to make inferences about the real potential of GG-FL to reduce the number of readers with dyslexia.

## Data Availability Statement

The raw data supporting the conclusions of this article will be made available by the authors upon request, without undue reservation.

## Ethics Statement

The studies involving human participants were reviewed and approved by the Medical Ethical Committee of UZ Leuven, KU Leuven. Written informed consent to participate in this study was provided by the participants' legal guardian/next of kin.

## Author Contributions

FVB conducted the statistical analysis, the data collection, project administration, data visualization, and wrote the original draft. ME and SVH were involved in the project administration and the review and editing part of the writing process. TG developed the software programs and was involved in the statistical analysis and review and editing part of the writing process. JV, MV, JW, and PG were involved in the project conceptualization, methodology, the review and editing part of the writing process, funding acquisition, and supervised the project. All authors contributed to the article and approved the submitted version.

## Funding

This study received funding from the Research Council of KU Leuven (C14/17/046). JV was a postdoctoral fellow of the Research Foundation Flanders (FWO) (12T4818N). These funding agencies independently stimulate and financially support research of clinical relevance.

## Conflict of Interest

The authors declare that the research was conducted in the absence of any commercial or financial relationships that could be construed as a potential conflict of interest.

## Publisher's Note

All claims expressed in this article are solely those of the authors and do not necessarily represent those of their affiliated organizations, or those of the publisher, the editors and the reviewers. Any product that may be evaluated in this article, or claim that may be made by its manufacturer, is not guaranteed or endorsed by the publisher.
